# Food-borne disease prevalence in rural villages in the Eastern Cape, South Africa

**DOI:** 10.4102/phcfm.v10i1.1796

**Published:** 2018-09-26

**Authors:** Khanya Z. Bisholo, Shanaz Ghuman, Firoza Haffejee

**Affiliations:** 1 Department of Community Health Studies, Durban University of Technology, South Africa; 2 Department of Basic Medical Sciences, Durban University of Technology, South Africa

## Abstract

**Background:**

The highest burden of food-borne diseases is in Africa. Despite this, food safety does not seem to be a major concern in many African countries. There is also a lack of concern within rural areas of South Africa.

**Aim:**

The aim of this study was to determine the prevalence of food-borne diseases in rural areas in the Eastern Cape, South Africa, by comparing data obtained from a cross-sectional survey and clinic records.

**Setting:**

The study was conducted in Ncera, Mpongo and Needscamp villages in the Eastern Cape, South Africa.

**Methods:**

In the first phase of the study, a random sample of household heads (*n* = 87) were interviewed to determine the prevalence of food-borne diseases between 2012 and 2014. In the second phase, registers from clinics serving the villages were screened for food-borne disease cases during the same time period.

**Results:**

A total of 109 (27.3%) household members fell ill because of food-borne diseases. Half (*n* = 56; 51.3%) of the respondents who fell ill because of food-borne diseases did not seek medical treatment for their illness. Of those who sought treatment, 19 (46%) attended primary health care clinics. However, examination of the clinic registers showed only four recorded cases of food-borne diseases.

**Conclusion:**

The prevalence of food-borne diseases in rural villages in the Eastern Cape, South Africa, was reported as high but the records in clinic registers are low, indicating a gap in the health care system. Monitoring of these diseases needs to improve.

## Introduction

Globally, one out of 10 people fall ill after consumption of contaminated food, with the highest burden occurring in Africa, followed by Southeast Asia, whereas in Europe, the lowest burden of food-borne diseases is reported.^1^ The prevalence of food-borne diseases varies vastly in different countries.^2,3,4,5,6^

In Africa, it is estimated that 92 million people fall ill from consuming contaminated foods, resulting in 137 000 deaths each year.^1,7^ Yet, food safety does not seem to be a major concern within many countries in this continent. For instance, in Ghana, Mali, Kenya and Uganda, food safety does not appear to be a major concern.^8,9,10,11^ In South Africa, Korsten^12^ states that there is no adequate capacity to forecast and track food-borne diseases even though there have been many outbreaks of food-borne diseases, across different provinces, particularly among school children.^13,14,15^ This indicates the necessity for a good surveillance system, which could monitor the outbreak of a food-borne disease and prevent it from spreading.^16^

In developed countries such as the United States, surveillance systems were developed to collect, analyse and share health data.^17^ However, many challenges of food safety exist in South Africa. These include a lack of coordination of the many government departments, which regulate food safety.^18,19^

Food-borne diseases is likely to increase in low- and middle-income countries because of the consumption of food such as uninspected meat, fish products as well as fresh produce.^20^

An effective epidemiological surveillance for food-borne diseases leads to better management and control of food-borne incidence. Malangu^21^ argues that food-borne diseases can be measured in terms of morbidity and mortality; nevertheless, because of a lack of data, it is impossible to estimate the mortality resulting from food-borne diseases in Africa. Furthermore, large proportions of ill people do not consult with a doctor or visit a health care facility.^22^

The surveillance of food-borne diseases is a critical component for food safety. Many of the food-borne disease outbreaks in South Africa have been reported by the media but are not recorded in an epidemiological surveillance system. This is the first epidemiological investigation to determine food-borne diseases in the Buffalo City Metro Health District (BCMHD) in the Eastern Cape province of South Africa. It is the centre of this largely rural province and is surrounded by many low-income and semi-rural villages, which are scattered across a mountainous landscape. In these villages, food is sold at many informal food outlets and street vendors. Livestock are also slaughtered at homesteads. The few clinics that serve these areas are scattered, and villagers often have to travel for more than 60 km to reach the clinic. The combination of the above factors in the community is a cause for public health concern.

This study determined the prevalence of food-borne diseases in the rural villages of Ncera, Mpongo and Needscamp, which form part of the BCMHD. We investigated this prevalence by a cross-sectional study among the residents of these villages as well as by an examination of clinic records. We thus also compared self-reported cases and health system records.

## Methods

### Study design

A retrospective, observational, quantitative design was used in this study.

### Settings

Buffalo City Municipality (BCMM) includes many low-income and semi-rural villages. These villages have more than 50 schools which prepare food on school premises. In BCMM, food is served at many informal shops situated in informal settlements and rural areas. These do not have certificates of acceptability, which is a legal prerequisite for all food handling premises in South Africa (Regulation, 962).

### Study population and sampling strategy

The sampling method occurred in two phases. In the first phase, a stratified random sample of household heads in the villages of Ncera, Mpongo and Needscamp, were interviewed for the study. Using the total population of 2 115 in the three villages, a 95% confidence level and 0.5% confidence interval, a minimum of 326 participants were required. As most of the households had more than four residents, a minimum of 87 households were calculated. The households were selected by random sampling. The house numbers were randomly chosen by a computer program to minimise sampling bias. The household heads in these selected households were invited to participate by completing a self-administered questionnaire.

All participants were adults, 18 years and older and residing in either Ncera, Mpongo or Needscamp village for more than six months. All participants were required to be fluent in either English or isiXhosa.

In the second phase of the study, purposive sampling of clinic records was conducted. The clinic registers at the Ncera, Mpongo and Needscamp clinics were examined to determine whether there were any patients who attended these clinics for food-borne diseases between 2012 and 2014.

### Data collection

In the first phase, a cross-sectional survey was conducted among household heads. Each household head was provided with a questionnaire in their language of preference (either English or isiXhosa). Those who were not able to read or write were assisted by the researcher; in such instances, the researcher asked the questions and the participants’ responses were scribed by the researcher onto the questionnaire. The questionnaire that was used for this study was adapted from that of a similar study in the United States. Permission was sought and granted by the authors of that study. Questions were structured into the following sections: demographic data, medical history of family members and information on food-borne diseases. The research tool was subjected to a strict interrogation by a group of five experts, comprising the researcher, the supervisor and three other academics from the field. The research questionnaire was then tested for accuracy in a pilot study that consisted of six respondents from the study population. This ensured validity and reliability of the questionnaire.

In the second phase, data were collected from 378 clinic registers, which were examined to extract data of patients who were treated for food-borne diseases.

### Data analysis

Quantitative data obtained were captured in a coded format on an Excel spreadsheet. The statistical package, SPSS version 23, was used for the statistical analysis of data. Frequencies of all categorical data were calculated. A chi-square test was used to determine the correlation between categorical variables.

### Ethical consideration

This research was approved by the Institutional Research Ethics Committee (IREC 058/16). Permission to conduct the study in the Eastern Cape was obtained from the Eastern Cape Department of Health. The Buffalo City Metropolitan Health District also endorsed the study. In all the households where data were collected, the researcher explained the purpose of the study to the head of the household in his or her preferred language, either English or isiXhosa. All respondents were informed that participation was voluntary and that they were allowed to withdraw at any stage of the study. No names or other identifiable information was collected on the questionnaire. The signed consent form was collected separately from the questionnaire, which was collected in a sealed ballot box.

## Results

### Phase 1

Data were obtained from three villages, namely Ncera, Needscamp and Mpongo. Twenty-nine household heads were from Ncera, 28 from Needscamp and 30 from Mpongo. In total, 87 household heads were interviewed in the three villages. In these households, there were a total number of 399 people. The demographic characteristics of the household heads are presented in Table 1. Over half of household heads were female (*n* = 51; 58.6%), only 33 (37.9%) were married and the majority were of the Black African race (*n* = 86; 98.8%). In general, education and income levels were low (Table 1). Only 24 (6.0%) of the total household members were HIV-positive. The majority did not report any other medical condition (*n* = 62; 71.3%). Most of the residents (*n* = 84; 96.5%) used the primary health clinic for their health care needs.

**TABLE 1:** Demographic characteristics of household heads.

Demographic factor	*n*	%
**Gender**		
Female	51	58.6
Male	36	41.4
**Marital status**		
Married	33	37.9
Single	25	28.7
Widowed	15	17.2
Living together	11	12.6
**HIV status**		
Negative	56	64.4
Positive	31	35.6
**Education level**		
Primary school	24	27.6
High school	39	44.8
Tertiary education	20	23
No formal education	4	4.6
**Monthly household income**		
Less than R1 200	13	14.9
R1 500–R3 500	45	51.7
R4 000–R6 000	24	27.6
R10 000–R15 000	1	4.6
More than R15 000	1	1.1
**Health facility used**		
Public clinic (free)	84	96.5
Private (paid by head of household)	1	1
Private (medical aid)	1	1.1

Note: All percentages do not total to 100 as some participants did not answer all questions.

A total of 109 (27.3%) household members fell ill because of food-borne diseases in Ncera, Mpongo and Needscamp villages. Vomiting was reported by 49 (44.9%) of these respondents, whereas both nausea and abdominal cramps were experienced by 37 (33.9%) of those who were infected. Most of the participants with food-borne diseases were between 35 and 49 years old.

Half (*n* = 56; 51.3%) of the respondents who fell ill because of food-borne diseases did not seek medical treatment for their illness. Of those who sought treatment, 19 (46%) participants reportedly sought treatment at primary health care clinics, which provided free treatment, and 2 (4.8%) participants sought medical treatment from private practitioners. Some used home remedies (*n* = 6; 14%) and others bought over the counter medication from the pharmacy (*n* = 5; 12.1%) to treat their condition. Some of the respondents who did not seek medical treatment used homemade remedies. The home remedies used by household heads were aloe mixtures (*n* = 2; 2.3%), camphor (*n* = 4; 4.6%), Coke (*n* = 2; 3%) and rooibos tea (*n* = 3; 3.4%). No traditional healer’s prescription was reported. Only 4 (9.7%) participants were requested to provide a stool sample for testing when they presented symptoms of food-borne illness, whereas 3 (7.3%) participants reported that the health care provider did nothing.

**FIGURE 1:**
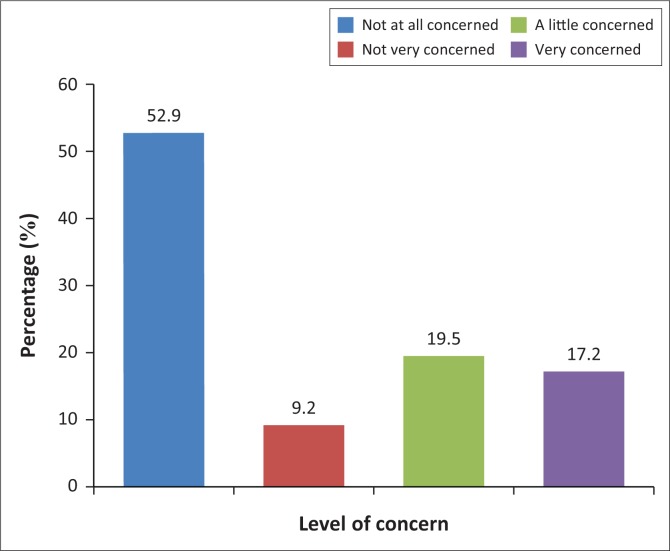
Food safety concern levels about food prepared away from home.

Figure 1 displays the percentage of food safety concern by household heads on food prepared away from home. Most household heads reported that they were not concerned at all about the safety of food prepared away from home (*n* = 46; 52.9%) and 8 (9.2%) participants were not very concerned.

**FIGURE 2:**
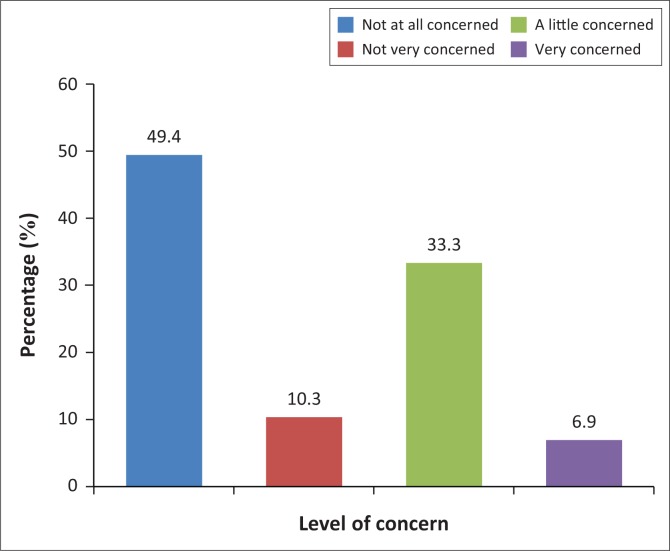
Food safety concern levels about food prepared at home.

In Figure 2, the percentage of those concerned about the safety of food at home is reported. Only 6 (6.9%) participants were very concerned about the safety of prepared food at home, whereas 43 (49.4%) participants were not concerned at all. The study found that the relationship between safety concern levels about food prepared at home and away from home was statistically significant (*p* < 0.001).

Most household heads reported that they store their perishable food in a refrigerator (*n* = 69; 79.3%). Only 9 (10.3%) households did not have a refrigerator. A small number of households (*n* = 4; 4.5%) buy food daily as required because they do not have either a refrigerator or electricity.

More than half of the household heads (*n* = 50; 57.4%) indicated that increased inspections to food outlets in the villages would reduce the risk of food-borne diseases. Another 16 (18.3%) household heads indicated that health education would reduce the risk. Less than a tenth (*n* = 7; 8.0%) indicated that increased monitoring programmes in food outlets were required, and 6 (6.9%) respondents reported that stiffer penalties for those food handlers who contravene health regulations would reduce the risk of food-borne diseases. A large number of household heads reported that they were not diagnosed with cholera when suffering from diarrhoea (*n* = 82; 94.2%) and only one (1.1%) person had diarrhoea while being ill with influenza. Most household heads in these villages reported that they wash hands with detergent before handling food (*n* = 78; 89.7%). Most households receive piped water (*n* = 67; 77.0%), although 15 (17.2%) of these households depended of water tanks on their premises. Five (5.7%) consumed water from a communal tap or tank.

A significant relationship existed between education levels and food safety concern levels (*p* < 0.001). People with higher education levels were more concerned about the safety of food prepared away from home. A large percentage of household heads, who did not have formal education or only had primary school education, were not concerned at all about the food safety of food that is prepared away from home or purchased from informal traders and supermarkets (*n* = 21; 75.0%). Those with some form of tertiary education (*n* = 20; 23%) were very concerned about the food safety of food prepared away from home. The relationship between the education level of household heads and their income was statistically significant (*p* = 0.03). There was thus a direct relationship between income levels and food safety concern (*p* < 0.001).

### Phase 2

A total of 378 clinic registers were scrutinised. The registers in the clinics, Mpongo (119), Ncera (124) and Needscamp (135), were scrutinised for the period 2012–2014. In Mpongo and Needscamp, there were no cases of reported food-borne diseases. However, there were four recorded cases of food-borne disease in Ncera clinic in 2012 and none in 2013 and 2014. The professional nurse in charge at the clinic indicated that they usually refer food-borne diseases to Frere Hospital in East London. Nevertheless, these cases did not appear in the transfer book that was used in 2012 and it was not clear whether these cases were notified.

## Discussion

The community survey indicated that more than a quarter of the household members who lived in these villages fell ill because of food-borne diseases during the period 2012–2014. Although 19 participants reported seeking treatment at a primary health care clinic for the food-borne disease, only four cases of food-borne diseases were recorded in the clinic registers during the same time period. This indicates a gap in the health surveillance system.

The participants reported food-borne symptoms, which confirmed their diagnosis. Wu et al. state that there is no single testing method for an intestinal pathogen.^23^ Health practitioners also rely on patients’ symptoms when making a diagnosis. Taege^7^ elaborates that careful history taking is important, followed by physical examination and laboratory evaluation. However, we report that only four stool specimens were taken for laboratory analysis to confirm diagnosis of a food-borne disease. Another study indicated that health care professionals focused on treatment of symptoms of the illness rather than confirmation of the diagnosis by laboratory testing.^24^ They were also not concerned about the reporting of notifiable conditions.^24^

This study reported that more than half of the people who fell ill because of food-borne diseases in these villages did not seek medical treatment for their illness. According to Hussain et al.,^25^ it is a common practice that many people do not seek medical help for food-borne illnesses. Furthermore, a study conducted in Guyana, South America, also found that 76.7% of cases of gastroenteritis were unreported. Health officials are concerned about this under-reporting and suggested that this can be improved with education.^24^ Other studies estimated that the reported incidence of food-borne diseases represents 1% of the actual incidence.^26^ Furthermore, Frean^27^ states that food-borne diseases are under-reported and poorly investigated in South Africa.

It is interesting to note that more than half of the household heads (52.9%) were not at all concerned about the safety of food prepared away from home. Despite this, the majority (57.4%) felt that increased inspections of food outlets were required to decrease food-borne diseases. Thus, the need for monitoring systems is recognised and most certainly required. Previous findings indicate that a higher number of food-borne disease outbreaks are associated with food prepared away from home.^28^ The BCMM report in 2016 showed that more than 65% of informal traders and food outlets in rural areas do not adhere to health standards, nor do they have certificates of acceptability as required by the South African legislation.^29^

It is noteworthy that people with higher education levels were concerned about the safety of food prepared away from home. This finding is corroborated by a similar study that was conducted in Mexico.^30^ We thus propose that in addition to increasing monitoring systems of prepared food, health education in the community to raise awareness of food-borne diseases is required. More health awareness in rural communities is needed for safer food preparation in rural settings. This can be achieved through school roadshows, education in clinics and posters in food premises. We also suggest that the ward encourages health-seeking behaviours in adults during their routine meetings with local authorities.

## Conclusion

The prevalence of food-borne diseases in rural villages in the Eastern Cape, South Africa, is high, but the records in clinic registers are low and do not tally with the self-reported incidence of these diseases. We recommend that better surveillance systems be developed, particularly in rural communities of South Africa. We also recommend that better monitoring systems for food outlets be developed and that health education awareness campaigns regarding food safety be conducted in rural areas.

### Limitations of the study

This was a retrospective study that required participants to respond to questions from recall, which may have affected the results to some extent. Future studies that are conducted close to the time of an outbreak would be more useful as those would determine which food was consumed to cause the outbreak. These studies should also test both food and stool samples for pathogens to confirm the diagnosis of food-borne diseases.
